# Methicillin-resistant and methicillin-sensitive *Staphylococcus aureus* in pork industry workers, Catalonia, Spain

**DOI:** 10.1016/j.onehlt.2023.100538

**Published:** 2023-04-07

**Authors:** Sara Quero, Marina Serras-Pujol, Noemí Párraga-Niño, Carmen Torres, Marian Navarro, Anna Vilamala, Emma Puigoriol, Javier Diez de los Ríos, Elisenda Arqué, Judit Serra-Pladevall, Alba Romero, Daniel Molina, Roger Paredes, Maria Luisa Pedro-Botet, Esteban Reynaga

**Affiliations:** a Infectious Diseases Unit, Health Sciences Research Institute of the Germans Trias i Pujol Foundation, Badalona, Barcelona, Spain; b CIBER de Enfermedades Respiratorias, Madrid, Spain; c Internal Medicine Department, Hospital Universitari de Vic, Barcelona, Spain; d Department of Medicine, Universitat Autònoma de Barcelona, Barcelona, Spain; e Area de Bioquímica y Biología Molecular, Universidad de La Rioja, Logroño, Spain; f Microbiology Department, Hospital Universitari de Vic, Barcelona, Spain; g Epidemiology Department, Hospital Universitari de Vic, Barcelona, Spain; h Fundació Lluita Contra les Infeccions, Infectious Diseases Department, Hospital Universitari Germans Trias i Pujol, Badalona, Barcelona, Spain; i IrsiCaixa AIDS Reseach institute, Hospital Germans Trias i Pujol, Badalona, Barcelona, Spain; j CIBERINFECT, ISCIII, Madrid, Spain

**Keywords:** *Staphylococcus aureus*, MRSA, LA-MRSA, MRSA-ST398, Spain, Pork industry workers

## Abstract

**Background:**

Methicillin-resistant *S. aureus* (MRSA) especially ST398, is a zoonotic agent. This study aimed to determine the prevalence of methicillin-susceptible *S. aureus* (MSSA) and MRSA among workers in the pork production chain.

**Methods:**

659 workers associated with 123 pig farms, livestock transporters, one pig slaughterhouse, pork transporters and 23 pork butcheries were studied for *S. aureus* recovery, and all isolates were characterized (antibiotic resistance, MLST and *spa*-typing).

**Results:**

The prevalence of *S. aureus* was 35.5%, 75.6% of isolates being MRSA. The prevalence of MRSA was 68.7% (149/217) among pig farm, 33.9% (19/56) livestock transporters, 2.9% (9/306) slaughterhouse, 0% in pork transporters (0/36) and butchery workers (0/44). Of the 234 *S. aureus*-positive workers, 100% (149/149) of pig farm workers, 82.6% (19/23) of livestock transporters, and 16.4% (9/55) of slaughterhouse workers carried MRSA isolates (*p* < 0.001). Of the workers who had contact with live swine, 61.8% (178/288) were *S. aureus*-positive, MRSA being detected in 96.1% of cases (p < 0.001). The most frequent lineage among MRSA were: ST398 (97.7%; 173/177) and ST1 (1.7%; 3/177); and among MSSA were ST30 (19.2%; 11/57) and ST5 (10.5%; 6/57). The most frequent *spa*-types among MRSA were t011 (93.8%, 166/177) and t1451 (2.25%, 4/177), and among MSSA: t084 (10.5%, 6/57) and t021 (7.0%, 4/57). All MRSA isolates showed resistance to tetracycline, 92.7% to clindamycin, 81.9% to erythromycin and 40.1% to cotrimoxazole.

**Conclusions:**

Pig industry workers having occupational contact with live animals present a high risk of colonization of MRSA, especially by MRSA-ST398. Prevention measures should be intensified in any employment sector involving live animals.

## Highlights

• Pig industry workers having occupational contact with live animals present a high risk of colonization of MRSA**.**
• In the *S. aureus* strains detected, the most frequent lineage was ST398.
• The most frequent *spa* type among MRSA isolates was t011.
• Most of the LA-MRSA strains presented a multidrug-resistant phenotype.

## 1 Introduction

Livestock-associated methicillin-resistant *Staphylococcus aureus* (LA-MRSA) of the lineage ST398 is a zoonotic agent, that primarily affects people who work with livestock or otherwise have contact with animals [1]. However, infections have also been reported occasionally in people who had no contact with animals [2]. Although MRSA ST398 colonization of occupationally exposed persons is most often asymptomatic, it can pose a risk to farmers, veterinarians, butchers and other exposed personnel as well as their household contacts because they can also be opportunistic pathogens [3]. Work stations in slaughterhouses, at least at the initial stage of meat processing which involves the handling and slaughter live animals, are ideal sites for measuring the prevalence of MRSA among plant personnel [4].

LA-MRSA is widespread in pigs, and MRSA ST398 is the most frequently reported genetic lineage on European pig farms [5]. Catalonia is the region of Spain with the biggest pig industry and thus represents one of the highest density pig populations per km^2^ in the country. Pig fattening farms are particularly concentrated in the area of Osona, and employment in the sector is very high representing a risk for MRSA ST398 colonization and infection [6,7]. However, there are no data available on the prevalence of *S. aureus* carriage at each of the points making up the pork production chain in this area. The present study seeks to partly fill this gap by analyzing the prevalence of *S. aureus* in pork workers in Osona, determining the differences among the strains detected, and analyzing the risk of MRSA carriage for pork industry employees at the various stages of the production chain.

## 2 Methods

We conducted a cross-sectional prevalence study in the county of Osona Catalonia, Spain, from June 2019 to December 2019. Epidemiological data gathered from pork industry employees including age, sex, years worked in the sector, and specific role within the production chain were cross-checked against evidence of colonization by *S. aureus* strains collected by nasal swab. The project was approved in advance by the research ethics committee of our institution, the Hospital Universitari de Vic.

### 2.1 Participating workers

Data was gathered during in-person visits to a total of 123 farms, Live animal transport workers, one pig slaughterhouse, pork transport workers and 23 pork butcheries in different localities of the Osona region. All workers over the age of 18 who were present at the time of the visit were invited to participate in the study, and all participants signed informed consent forms. In order to include as many of the 400 workers at the slaughterhouse as possible, the site was visited during the morning, afternoon and night shifts. Live animal transport workers were swabbed at the entrance of the slaughterhouse just before unloading the pigs, and pork transport workers were swabbed as they loaded finished pork products at the exit of the slaughterhouse.

### 2.2 Production process in the slaughterhouse

Around 3000 live pigs arrive from pig farms at the slaughterhouse every day. When the pigs arrive, they are first collected in the lairage area, where they are calmed with a shower of water and then allowed to rest for 2 h. After the animals are stunned with carbon dioxide, the carotid artery is cut, causing them to bleed to death. Next, the carcasses are scalded and depilated and the internal organs are removed in the scalding dehairing and evisceration area. After the eviscerated carcasses are quartered, cut and boned in the cutting area, the meat goes to the processing and packing area, where it is processed into smaller cuts and packaged. Finally, in the shipping area, the meat is loaded onto road transport for shipping to local, national or foreign butcheries.

### 2.3 Collection and processing of samples

A nasal swab was collected from both nostrils of each participant with cotton-tipped swabs that were then placed in Stuart swab PS+ Viscose (Deltalab, Rubí, Spain). The swabs were stored at 4 °C and transported directly to the Microbiology Department of the Hospital Universitari de Vic for analysis.

#### 2.3.1 MRSA and MSSA isolation and characterization

*S. aureus* strains were identified by common standard microbiological assays including catalase, Gram staining, DNase and coagulase [8]. The samples were cultured on Brilliance MRSA chromogenic agar (Oxoid, PO5196A, UK) and the results were read after 48 h. Suspected colonies (green) were plated onto blood agar (Oxoid, CM0055, UK), and organism identification was performed using an automated system (bioMérieux Vitek 2). For the confirmation of MRSA isolates, susceptibility to oxacillin and cefoxitin was determined by a disk diffusion test (9), and the presence of the PBP2a protein was analyzed by a latex agglutination test with specific anti-PBP2 monoclonal antibodies (Slidex® MRSA detection, bioMérieux). MRSA strains showed resistance to oxacillin and cefoxitin and were positive for PBP2a protein in the agglutination test.

#### 2.3.2 Antibiotic susceptibility testing

Susceptibility testing was carried out by the disk diffusion method following the recommendations of the European Committee on Antimicrobial Susceptibility Testing (EUCAST) [9]. Isolates were maintained at −80 °C for other determinations.

#### 2.3.3 DNA extraction and quantification

DNA extraction of *S. aureus* strains was performed using the QIAamp DNA Blood Mini Kit (QIAGEN, Hilden, Germany) following the manufacturer instructions. Extractions were quantified in QubitTM fluorometer (Invitrogen, Waltham, USA).

#### 2.3.4 Whole genome sequencing

DNA extractions were normalized at 0.2 ng/μl for library preparation with Nextera XT DNA Library Preparation Kit (Illumina, San Diego, USA). After amplification step, the samples were purified with CleanNGS beads (CleanNA, Waddinxveen, Netherlands). QC of libraries was done in a TapeStation 2200 (Agilent). Libraries were individually quantified by fluorimetry (Quantus, Promega), pooled and run in a MiSeq (at 10pM final concentration containing 10% PhiX) and 100.000 K reads per sample were obtained. Sequencing was performed at the Genomic Core Facility at Germans Trias i Pujol Research Institute.

Raw sequences were imported as paired-end sequences in KBase platform [10]. Reads were de novo assembled using SPAdes assembler app v3.13.0, with default parameters, to obtain contigs in FASTA files. These contigs were used to genotype *S. aureus* isolates using Multi Locus Sequence Typing (MLST) [11] and spaTyper [12] tools from Center for Genomic Epidemiology (Technical University of Denmark).

### 2.4 Statistical analysis

Statistical analysis was performed using IBM SPSS Statistics software version 28.0. Qualitative variables were expressed as frequency and percentages and quantitative variables as mean and standard deviation (SD). The chi-square test (Fisher's exac*t-*test) and the Student's *t-*test were used to compare epidemiological characteristics between those participants colonized by *S. aureus* and those not colonized, and also between MRSA-carrying and MSSA-carrying participants. In order to identify factors associated with MSSA and MRSA colonization, a univariate logistic regression was performed and subsequently with the univariate factors associated, multivariate logistic regressions was carried out. Statistical significance was set at *p* < 0.05.

## 3 Results

Data was obtained from a total of 659 workers in the pork production chain, consisting of 217 pig farm workers, 56 live animal transporters, 306 slaughterhouse workers, 36 pork transporters and 44 butchery workers. The mean age was 43.1 (SD 13.7) years and 71.2% (469) were male. The mean number of years worked was 13.4 (SD ± 11.3).

The prevalence of *S. aureus* colonization among all participants was 35.5% (234/659). By workplace, colonization was detected in 68.7% (149/217) of pig farm workers, 41.1% (23/56) of Livestock transporters, 18.0% (55/306) of slaughterhouse workers, 8.3% (3/36) of pork transporters and 9.1% (4/44) of butchery workers (*p* < 0.001).

**Fig. 1:**
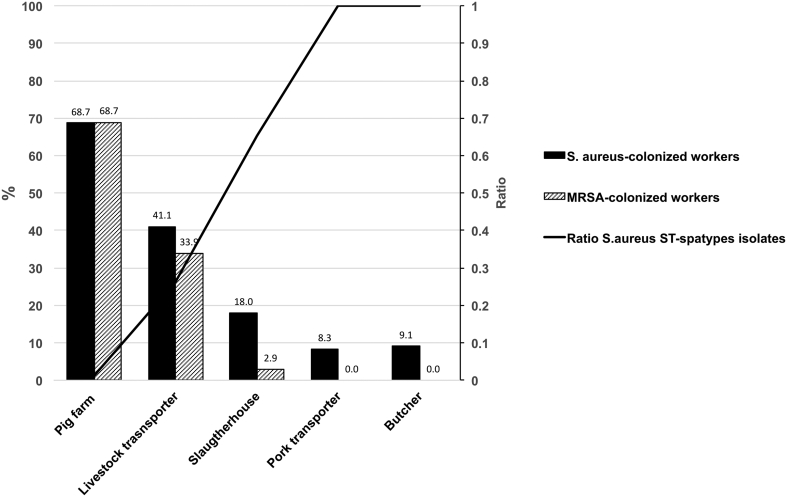
Prevalence of MRSA and MSSA among the *S. aureus*-colonized workers and ratio ST-spa type in the pork production chain.

Of the 234 *S. aureus*-positive workers, 75.6% (177/234) carried MRSA isolates. Among *S. aureus*-positive workers, MRSA was found in 100% of pig farmers (149/149), 82.6% of live animal transporters (19/23) and 16.4% of slaughterhouse workers (9/55), while MSSA was detected in 100% of pork transporters (3/3) and butchers (4/4) (*p* < 0.001) (Fig. 1).

The prevalence of MRSA by workplace was 68.7% (149/217) in farmers, 33.9% (19/56) in live animal transporters, 2.9% (9/306) in slaughterhouse workers, 0% (0/36) in pork transporters and 0% (0/44) in butchery workers (Table 1*).*

**Table 1:** Prevalence of MRSA in workers at various stages of the pork production chain.

Characteristics		Non MRSA Δ n (%)	MRSA n(%)	p	OR (95% CI) No *S. aureus* + MSSA – MRSA
All workers		482 (73.1%)	177 (26.9%)		
Contact with pig	No	28 (100.0%)	0 (0.0%)	**0.001**	
	Yes	453 (71.9%)	177 (28.1%)		
Contact with live pig	No	365 (98.4%)	6 (1.6%)	**<0.001**	1
	Yes	117 (40.6%)	171 (59.4%)		54.0 (23.1–126.3)
Gender	Male	314 (67.0%)	155 (33.0%)	**<0.001**	
	Female	168 (88.4%)	22 (11.6%)		
Age (SD±)		41.7 ± 12.4	47.6 ± 16.5	**<0.001**	
Years worked (SD±)		12.7 ± 10.9	16.6 ± 12.7	0.001	
Pig farm worker		68 (31.3%)	149 (68.7%)		
Livestock transport worker		37 (66.1%)	19 (33.9%)		
Slaughterhouse*		297 (97.1%)	9 (2.9%)	**<0.001**	
Pork transport worker		36 (100.0%)	0 (0.0%)		
Butcher		44 (100.0%)	0 (0.0%)		
Slaughterhouse*					
Lairage area		8 (72.7%)	3 (27.3%)		
Scalding, dehairing and evisceration area		40 (95.2%)	2 (4.8%)		
Cutting area		137 (97.9%)	3 (2.1%)		
Processing and packing area		52 (100.0%)	0 (0.0%)		
Shipping area		23 (95.8%)	1 (4.2%)		
Technical service		9 (100.0%)	0 (0.0%)		
Other ^		28 (100.0%)	0 (0.0%)		

Δ Non MRSA: Negative *S. aureus* + MSSA.

^ Other: Laundry, laboratory, offices and veterinarians.

Of the workers having contact with live swine, 43.7% (288/659) were *S. aureus*-positive, the strain involved being MRSA in 96.1% of cases (*p* < 0.001). Table 2 summarizes the epidemiological characteristics and prevalence of *S. aureus* colonization in the workers analyzed and compares the prevalence of MRSA vs. MSSA colonization among the different jobs in the pork production chain. Moreover, the prevalence of MRSA was higher among males than females (33%; 171/469 versus 11.6%; 22/190, *p* < 0.001). The 22 female workers colonized with MRSA, consisted of 19 (90.4%) of the 21 female pig farm workers and 3 of 133 female slaughterhouse workers.

**Table 2:** Prevalence and Epidemiological characteristics of *S. aureus* at various stages of the pork production chain.

Characteristics		*S. aureus* n (%)	No *S aureus* n (%)	p	OR (95% CI) *S. aureus* – No *S. aureus*	MSSA n (%)	MRSA n (%)	p	OR (95% CI) MSSA - MRSA
All workers		234 (35.5%)	425 (64.5%)			57 (24.4%)	177 (75.6%)		
Contact with pig	No	7 (25.0%)	21 (75.0%)	<0.233		7 (100.0%)	0 (0.0%)	**<0.001**	
	Yes	227 (36.0%)	403 (64.0%)			50 (22.0%)	177 (78.0%)		
Contact with live pig	No	56 (15.1%)	315 (84.9%)	**<0.001**	1	50 (89.3%)	6 (10.7%)	**<0.001**	1
	Yes	178 (61.8%)	110 (38.2%)		3.29 (1.12–9.67)	7 (3.9%)	171 (96.1%)		203.6 (65.4–633.4)
Gender	Male	192 (40.9%)	277 (59.1%)	**<0.001**		37 (19.3)	155 (80.7)	**<0.001**	
	Female	42 (22.1%)	148 (77.9%)			20 (47.6%)	22 (52.4%)		
Age (SD±)		46.0 ± 15.7	41.7 ± 12.5	**<0.001**		41.2 (12.1)	47.6 (16.5)	**0.007**	
Years worked (SD±)		14.5 ± 11.8	13.0 ± 11.1	0.142		10.7 (8.7)	16.6 (12.7)	**0.002**	
Pig farm worker		149 (68.7%)	68 (31.3%)		6.07 (1.33–27.70)	0 (0%)	149 (100.0%)		
Livestock transport worker		23 (41.1%)	33 (58.9%)		2.12 (0.44–10.29)	4 (17.4%)	19 (82.6%)		
Slaughterhouse*		55 (18.0%)	251 (82.0%)	**<0.001**	2.03 (0.69–5.92)	46 (83.6%)	9 (16.4%)	**<0.001**	
Pork transport worker		3 (8.3%)	33 (91.7%)		0.91 (0.19–4.35)	3 (100.0%)	0 (0.0%)		
Butcher		4 (9.1%)	40 (90.9%)		1	4 (100.0%)	0 (0.0%)		

SLAUGHTERHOUSE*									
Lairage area		5 (45.5%)	6 (54.5%)			2 (40.0%)	3 (60.0%)		
Scalding, dehairing and evisceration area		5 (11.9%)	37 (88.1%)			3 (60.0%)	2 (40.0%)		
Cutting area		20 (14.3%)	120 (85.7%)			17 (85.0%)	3 (15.0%)		
Processing and packing area		6 (11.5%)	46 (88.5%)			6 (100.0%)	0 (0.0%)		
Shipping area		10 (41.7%)	14 (58.3%)			9 (90.0%)	1 (10.0%)		
Technical service		4 (44.4%)	5 (55.6%)			4 (100.0%)	0 (0.0%)		
Other ^		5 (17.9%)	23 (82.1%)			5 (100.0%)	0 (0.0%)		

^ Other: Laundry, laboratory, offices and veterinarians.

### 3.1 MLST and phenotype antibiotic resistance

In the *S. aureus* strains detected, the most frequent lineage was ST398 (74.3%; 174/234). The most frequent MSSA lineage was ST30 (19.2%; 11/57), followed by ST5 (10.5%; 6/57). The lineages identified among MRSA isolates were ST398 (97.7%; 173/177), ST1 (1.7%; 3/177) and ST121 (0.6%; 1/177). The most frequent *spa* type among MRSA isolates was t011 (93.8%, 166/177), the other *spa* types detected being t1451 (2,25%, 4/177), t1456 (1,7%, 3/177), t1491 (1.1% 2/177), t127 (1/177) and t4190 (1/177). A great diversity of *spa* types was detected among MSSA isolates, including one ST398-t571 isolate. Table 3 shows the distribution of MLST and *spa* types according to the step in the pork production chain where the workers were employed and Fig. 1 shows the ratio of ST-spa type in the pork production chain.

**Table 3:** MLST and *spa* types isolated from all workers in the pork production chain.

	MRSA	Spa type	MSSA	Spa type
	(n)	(n)	(n)	(n)
Pig farm workers	(148) ST398	(148) t011		
	(1) ST121	(1) t4198		
Livestock transport worker	(19) ST398	(14) t011, (2) t1451, (3) t1456	(3) ST30	(2) t021, (1) t012
			(1) ST9	(1) t1430
Slaughterhouse				
Lairage area	(2) ST398	(2) t011	(2) ST25	(2) t349
	(1) ST1	(1) t1491		
Scalding, dehairing and evisceration area	(2) ST398	(2) t011	(1) ST5	(1) t002
			(1) ST30	(1) t021
			(1) ST45	(1) t550
Cutting area	(2) ST1	(1) t127, (1) t1491	(2)ST5	(1) t002, (1) t005
	(1) ST398	(1) t1451	(2) ST30	(2) t012
			(2) ST109	(1) t9885, (1) t209
			(1) ST672	(1) t3841
			(1) ST22	(1) t2833
			(1) ST398	(1) t571
			(1) ST1024	(1) t238
			(1) ST34	(1) t166
			(1) ST291	(1) t1149
			(3) ST1	(3) t1491
			(1) ST146	(1) t002
			(1) ST199	(1) t084
Processing and packing area			(1) ST582	(1) t084
			(2) ST30	(1) t012, (1) t021
			(1) ST5	(1) t311
			(1) ST15	(1) t084
			(1) ST3758	
Shipping area	(1) ST398	(1) t1451	(1) ST199	(1) t084
			(1) ST8	(1) t008
			(1) ST30	(1) t012
			(2) ST25	(1) t078, (1) t436
			(1) ST97	(1) t297
			(1) ST6	(1) t5477
			(1) ST2025	(1) t230
			(1) ST34	(1) t136
Technical service			(1)ST30	(1) t012
			(1) ST1842	
			(1) ST45	(1) t020
			(1) ST199	(1) t084
Other			(2) ST5	(1) t005
			(3) ST22	(1) t2833, (1) t310, (1) t005
Pork transport worker			(1) ST34	(1) t2485
			(1) ST72	(1) t148
			(1) ST30	(1) t2485
Butcher			(1) ST15	(1) t084
			(1) ST9	(1) t1430
			(1) ST45	(1) t020
			(1) ST1	(1) t1491

**Fig. 2:**
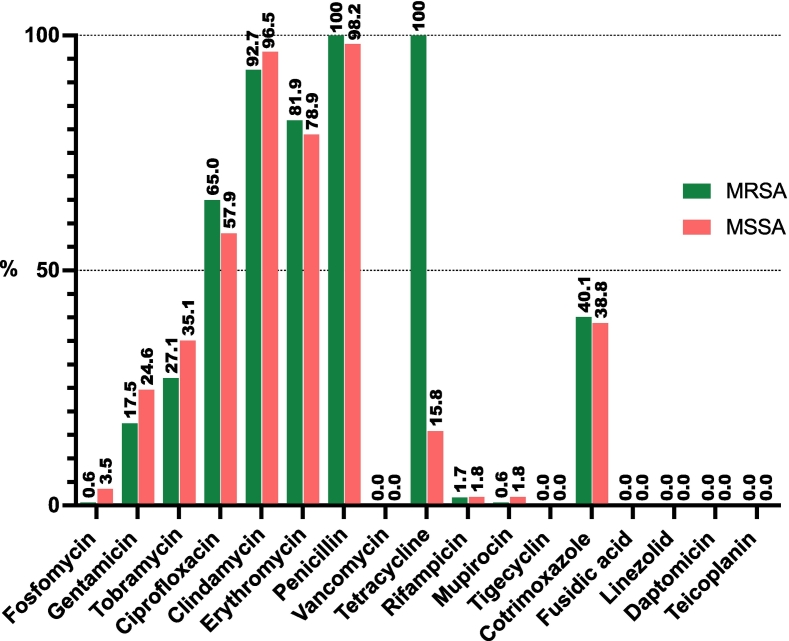
The phenotype of antibiotic resistance of *S. aureus* isolates (MSSA and MRSA) in workers.

All MRSA isolates showed resistance to tetracycline, 92.7% showed resistance to clindamycin, 81.9% to erythromycin and 40.1% to cotrimoxazole (TMX). All MRSA isolates were susceptible to vancomycin, daptomycin, linezolid, mupirocin and rifampicin. The phenotype of antibiotic resistance of *S. aureus* isolates (MSSA and MRSA) in workers is shown in Fig. 2.

## 4 Discussion

This is the first study in Spain that analyzes workers in the entire chain of the pork industry, from the pig farm worker to the butcher shop employee. Our results confirm that working with live pigs is a risk factor for being a carrier of MRSA, a microbe which, though part of the normal microbiota present in humans, may under certain circumstances cause disease, sometimes with serious consequences.

When comparing workers who work with live animals including pig farm workers, live animal transporters and lairage area workers to those whose contact is limited to pork meat, including other slaughterhouse workers, pork transporters and butchers, it can be observed that the former have a higher probability of being MRSA carriers than the latter. In addition, it can be seen that as meat-processing progresses down the chain, there is a clear decrease in MRSA colonization and an increase in MSSA colonization. Thus the prevalence of MRSA in pig farm work is clearly higher compared to slaughterhouse or even butchery work. In addition, MRSA colonization is possibly related to differences between the sorts of jobs in which males and females are engaged. The activities where males predominate are in pig farms and livestock transporters workers and therefore involve contact with live animals, while females are more likely to have jobs in slaughterhouses or butcheries that involve contact with meat, not live animals. In our study, the prevalence of MRSA in workers who have contact with live animals was 59.3%, which was higher than previous studies in the Netherlands and Italy, which noted a prevalence of 15.1% [13] and 17.3% [14], respectively.

The 68.7% prevalence of MRSA among pig farm workers reported in this study is comparable to the prevalence reported in similar studies carried out in the Netherlands (63%) [15] but much higher than the 25% reported from a region of Germany [16]. And alarmingly, the prevalence we report here is about 12% higher than that seen in a similar study performed by our group in the same area 5 years previously. In this study, 140 workers from 83 pig farms were analyzed, and 57.9% were MRSA positive; all of them were ST398 [6].

In another Dutch study, the prevalence of MRSA was shown to be 22% among live pig transport workers [13], which is lower than the 33.9% prevalence among analogous personnel reported in the present study, and still lower than that for pig farm workers, albeit much higher than the prevalence of MRSA in the general healthy human population in Spain (<0.5%) [17].

With regard to slaughterhouse work, the prevalence of MRSA was 2.9% overall, but for workers in the lairage area, who have most contact with live animals, the rate of colonization was much higher (27.2%). MRSA carriers were detected in other areas of the slaughterhouse, although one worker in the cutting area and one in the shipping area also worked on pig farms, and it was not possible to pinpoint the origin of the colonization. Similar data were found in a previous Dutch study that observed MRSA colonization in 3% of workers in three slaughterhouses overall; when they broke the figure down by zone, however they also observed that the lairage area (11%) was the slaughterhouse zone with the highest prevalence of MRSA [5].

No MRSA carriage was detected among pork transporters and butchery workers who only handle pork meat after it leaves the slaughterhouse. In addition to the fact that such occupations do not involve working with live animals, current meat handling regulations techniques, with mandatory hand hygiene and the use of gloves during working hours, may contribute to the low carrier status in these workers.

With regard to the *S. aureus* strains encountered, ST398 was the most prevalent lineage detected overall, and especially among MRSA isolates; this lineage was detected in 97.7% of all MRSA carriers. MRSA ST398 is reported to be the most prevalent pig-associated genotype in several countries, though other genotypes of MRSA typically associated with animals (ST97, ST9, ST1) or humans (ST5) have also been detected [14]. Our study identified several *spa* types among MRSA-ST398 isolates, with t011 predominating. This finding is consistent with other European surveys of pigs and pig farm workers [5,13,18,19].

In our study, a wide diversity of Spa types were present at different stages of the pork production chain. Pig farm workers who had contact with live pigs showed a high colonization rate but the diversity of ST-spa types was shallow, with only two different ST-spa types being present in the 149 isolates. On the other hand, this diversity increased down the production chain, so that in pork transporters and butchers each isolated *S. aureus* exhibited a different ST-spa-type. These findings reinforce the possibility that the increased colonization of workers is related to contact with live animals.

In the final steps of the slaughterhouse process, *S aureus* colonization was low and the high ST-spa-types diversity was similar to that observed in pork transporters and butchers. This seems to indicate that once the pig is dead, eviscerated, and cleaned, transmission of *S. aureus* between animal and person no longer takes place, and the *S. aureus* identified are common to those found in the general human population [17].

Most of the LA-MRSA strains presented a multi-resistant phenotype (resistance to at least three different families of antibiotics), including in all cases tetracycline resistance. The extensive use of tetracycline in pig farming has contributed to the selection of LA-MRSA in this species [20]. For this reason, some authors recommend using tetracycline resistance as a marker of LA-MRSA [21]. However, the continuous evolution of this organism and combined with the use of other classes of antimicrobials in the pig production chain may have led to the selection of strains that have modified their sensitivity pattern to antimicrobial agents [5].

With regard to the antibiotic resistance phenotypes in *S. aureus* isolates, resistance was especially high to clindamycin, which exceeded 90%, but it was also high to erythromycin (around 80%) and cotrimoxazole (40%) in both MRSA and MSSA. A similar resistance profile was identified in a previous study on pig farm workers, which pointed to an association with an enrichment in clindamycin resistance genes (especially *lnuA, lunB* and *vga*) among MRSA ST398 isolates [6].

In this study one MSSA-ST398 strain of *spa* type t571 was also identified. This isolate was recovered from personnel of the slaughterhouse cutting area. It must be highlighted that the MSSA-ST398-t571 lineage corresponds to the livestock-independent ST398 clade that has been increasingly reported in invasive human infections [22], but it is different from the livestock-associated MRSA-ST398 clade mostly detected in this study.

This study has several limitations. The first is that data from only one slaughterhouse were analyzed, and it would be risky to extrapolate our findings to other slaughterhouses, although it is true that all slaughterhouses in Spain must comply with the same hygiene regulations. A second limitation is that our data do not include workers involved in cleaning pig intestines, an operation which is in this particular instance outsourced to a separate company. Finally, environmental samples were not taken from the various workplace sites and analyzed, this issue being the subject of a future analysis.

Despite these shortcomings, however, we believe the analysis undertaken here constitutes a valuable contribution to what we know about the relationship between MRSA and the livestock industry, particularly the pig and pork industry.

## 5 Conclusion

Our data provide evidence that live animal workers have a high risk of being carriers of MRSA, especially MRSA ST398. By contrast, for workers who handle pork meat, rather than livestock, the chances of being a carrier are relatively low. Our findings suggest that in order to reduce these risks, prevention measures should be intensified at all stages of pork production process.

## Role of the funder/sponsor

The funder of the study had no role in study design, data collection, data analysis, data interpretation, or writing of the report.

## Financial support

This work was supported by the 10.13039/501100004587Instituto de Salud Carlos III, the 10.13039/501100004837Spanish Ministry of Science and Innovation (grant number PI18/01258).

## CRediT authorship contribution statement

**Sara Quero:** Conceptualization, Methodology, Validation, Formal analysis, Data curation, Investigation, Software, Writing – original draft, Writing – review & editing. **Marina Serras-Pujol:** Validation, Supervision, Data curation, Formal analysis, Writing – original draft, Writing – review & editing. **Noemí Párraga-Niño:** Methodology, Formal analysis, Writing – original draft, Writing – review & editing. **Carmen Torres:** Methodology, Formal analysis, Writing – original draft, Writing – review & editing. **Marian Navarro:** Validation, Formal analysis, Funding acquisition, Writing – original draft, Writing – review & editing. **Anna Vilamala:** Validation, Formal analysis, Funding acquisition, Writing – original draft, Writing – review & editing. **Emma Puigoriol:** Validation, Formal analysis, Funding acquisition, Writing – original draft, Writing – review & editing. **Javier Diez de los Ríos:** Validation, Formal analysis, Funding acquisition, Writing – original draft, Writing – review & editing. **Elisenda Arqué:** Validation, Formal analysis, Funding acquisition, Writing – original draft, Writing – review & editing. **Judit Serra-Pladevall:** Validation, Formal analysis, Funding acquisition, Writing – original draft, Writing – review & editing. **Alba Romero:** Validation, Formal analysis, Funding acquisition, Writing – original draft, Writing – review & editing. **Daniel Molina:** Validation, Formal analysis, Funding acquisition, Writing – original draft, Writing – review & editing. **Roger Paredes:** Validation, Formal analysis, Funding acquisition, Writing – original draft, Writing – review & editing. **Maria Luisa Pedro-Botet:** Validation, Formal analysis, Funding acquisition, Writing – original draft, Writing – review & editing. **Esteban Reynaga:** Conceptualization, Validation, Formal analysis, Data curation, Supervision, Project administration, Funding acquisition, Writing – original draft, Writing – review & editing.

## Declaration of Competing Interest

RP participated in advisory activities related to COVID-19 and HIV for Gilead, ViiV Healthcare, GSK, MSD, Theratechnologies, Lilly and Roche, and received research funds awarded to his institution from Gilead, ViiV healthcare and MSD. All other authors declare no competing interests.

## Data Availability

The data that has been used is confidential.
